# Anterior Chamber Angle Evaluation following Phakic Posterior Chamber Collamer Lens with CentraFLOW and Its Correlation with ICL Vault and Intraocular Pressure

**DOI:** 10.1155/2016/1383289

**Published:** 2016-12-08

**Authors:** Sherif A. Eissa, Sherin H. Sadek, Mohamed W. A. El-Deeb

**Affiliations:** ^1^Ophthalmology Department, Faculty of Medicine, Cairo University, Kasr Al Ainy, Cairo, Egypt; ^2^Ophthalmology Department, Faculty of Medicine, Fayoum University, Al Fayoum, Egypt

## Abstract

*Purpose.* To assess intraocular pressure (IOP), lens vaulting, and anterior chamber (AC) angle width, following V4C implantable Collamer lens (ICL) procedure for myopic refractive error.* Methods.* A prospective case series that enrolled 54 eyes of 27 patients that were evaluated before and after V4C phakic posterior chamber Collamer lens implantation for correction of myopic refractive error. Preoperative measurement of IOP was done using Goldmann applanation tonometer and anterior chamber angle width using both Van Herick slit lamp grading system and Scheimpflug tomography imaging (Oculus Pentacam). Follow-up of the aforementioned variables was at 1, 6, and 18 months postoperatively, together with ICL vault measurements.* Results.* The mean baseline IOP of 11.69 ± 2.15 showed a statistically significant (*P* = 0.002) increase after 1 month that remained unchanged at 6 and 18 months postoperatively, with mean value of 16.07 ± 4.12, 16.07 ± 4.10, and 16.07 ± 4.13, respectively. Pentacam AC angle width showed a statistically significant decrease at 1 (*P* = 0.025), 6 (*P* = 0.016), and 18 (*P* = 0.010) months postoperatively, with mean preoperative value of 40.14 ± 5.49 that decreased to 25.28 ± 5.33, 25.46 ± 5.44, and 25.49 ± 5.38, at 1, 6, and 18 months, respectively. Mean ICL vault showed moderate correlation with Pentacam AC angle width at 1 (*r* = −0.435) and 6 (*r* = −0.424) months.* Conclusion*. V4C ICL implantation resulted in decrease in AC angle width and increase in IOP, within acceptable physiological values at all time points.

## 1. Introduction

Many authors showed that conventional ICL insertion with peripheral iridotomy had no significant effect on postoperative IOP and that it resulted in a narrower angle width without increasing trabecular pigmentation (compared with values after laser iridotomy) [1].

The V4C model of ICL has got the KS-AquaPORT introduced to the center of the ICL optic, which improved aqueous humor circulation between posterior and anterior chambers. The nonoccludable 0.36 mm CentraFLOW diminished the risk of postoperative pupillary block that may occur following closure of peripheral surgical or laser iridotomy [2].

The distance between the posterior ICL surface and the anterior crystalline lens pole is termed the ICL vault, which is crucial regarding the incidence of anterior subcapsular cataract formation [3].

The ideal postoperative vault must create a space over the entire anterior crystalline lens surface, with 1.00 to 1.50 central corneal thickness (CCT), on slit lamp examination [4]. Whilst poor vault (<250 *μ*m) increases risk of cataract development, excessive vault (>750 *μ*m) may result in pupillary block-angle closure glaucoma [5].

ICL vault is determined by patient age, white-to-white (W-W) measurement [6], and ICL shape/design. The latter remains the most crucial factor in determining postoperative vault, with the V4 model resulting in higher vault, compared to the flatter V3 design ICL [5].

The central hole in V4C may affect the amount of pIOL vault, which plays a vital role in determining the safety of the pIOL implantation technique [7].

Nongonioscopic screening tests on limbal anterior chamber depth for the detection of occludable angles include Van Herick, ultrasound pachymetry, and optical pachymetry. Limitations of Van Herick include inter- and intraobserver variability in the measurement itself, lack of angle morphology description, and dependence on clear integral peripheral cornea [8].

Our study aims to evaluate the correlation between ICL power and vault, with postoperative IOP and anterior chamber angle width.

## 2. Patients and Methods

This prospective case series included 54 eyes of 27 patients with myopic refractive error. ICL implantation procedure was performed at Aseer Magrabi Eye Hospital, (MEH) in Kingdom of Saudi Arabia (KSA) in the period of December 2012 to November 2013. The mean age of the subjects was 29 ± 2.30 years. The ethical committee of MEH approved the study. Comprehensive discussion with the patients was undertaken before surgery, explaining for them the details of the procedure and its benefit and complications and an informed written consent was obtained from all patients. A single surgeon who is the first author performed all surgeries.

The inclusion criterion was myopic error with documented stable refraction for 12 months earlier. The exclusion criteria were an anterior chamber depth (ACD) less than 3.0 mm, history and/or clinical signs of iritis or uveitis, macular or retinal involvement, glaucoma or pigmentary dispersion, monocular vision, lens opacity, pseudoexfoliation, endothelial cell count less than 2500 cells/mm^2^, and W-W less than 11.00 mm.

All patients were evaluated for IOP using Goldmann applanation tonometer and anterior chamber angle width using both Van Herick slit lamp grading system and Scheimpflug tomography imaging (Oculus Pentacam). Follow-up of the aforementioned variables was at 1, 6, and 18 months postoperatively, together with ICL vault measurements.

All preoperative and postoperative investigations were performed at morning shift (9–12 a.m.) under mesopic-light illumination conditions.

The ICL vault was estimated clinically relative to CCT (1.00x) using slit beam, and the central vault of the pIOL over the crystalline lens was objectively evaluated with a rotating Scheimpflug camera (Pentacam HR, Oculus Optikgeräte GmbH) 1 month, 6 months, and 18 months postoperatively. Using the image analysis software program with this device, without cycloplegia, an experienced optometrist calculated the amount of the central vault of the pIOL over the crystalline lens as the distance between the posterior surface of the pIOL and the anterior surface of the crystalline lens.

Table 1 shows the Foster modification of the Van Herick grading system for limbal anterior chamber depth, using slit lamp biomicroscopy, applied in our study by the author [9].

Preoperative Scheimpflug imaging was mandatory in all cases, to measure the anterior chamber depth (ACD), central corneal thickness (CCT), anterior chamber angle width, and white-to-white (W-W) measurement.

We calculated the power of ICL (modified vertex calculation formula) by entering patients' ACD, manifest refraction spherical equivalent (MRSE), back vertex distance, and K readings on STAAR Company online calculator and ordering system (OCOS).

For the pIOL proper sizing, the system requires data entry by the user, for W-W measurement, ACD, CCT, beside the birth date, and any history of previous intervention like IOL implantation.

ICL size was determined by the manufacturer nomogram (OCOS) in all cases, without making any changes by the assigned surgeon. Mean W-W measurement was 11.7 ± 0.4 mm, and mean ICL diameter was 12.6 ± 0.5 mm.

All procedures were performed under topical anesthesia. ICL implantation was performed via the technique described by Alfonso et al. [10]. A 3.20 mm temporal tunneled clear cornea incision was created, and the anterior chamber was filled with viscoelastic material (Microvisc 1%; Bohus BioTech AB). The PC pIOL (Visian® ICL V4C; STAAR Surgical Inc., Monrovia, CA) was loaded into the cartridge and injected very slowly to allow controlled slow lens unfolding; then an iris manipulator was used to tuck the footplate haptics of the lens within the posterior chamber. The viscoelastic material was then removed using the Simcoe irrigation aspiration cannula. No peripheral iridectomy was needed, as KS-AquaPORT ensures dynamic regular aqueous flow between posterior and anterior chambers. Postoperatively, patients were prescribed gatifloxacin 0.5% eye drops (Zymar®, Allergan, Inc., Fort Worth, Texas) four times daily and prednisolone acetate 1% (Pred Forte® Allergan, Inc., Irvine, CA, USA) four times daily for 2 weeks.

Statistical analysis: SPSS 15 software was used for data analysis. Data were presented as mean ± SD for normally distributed data and medians (quartiles) for abnormally distributed data. Variables were analyzed in relation to baseline values using analysis of variance (ANOVA) for repeated measures. Pearson correlation analysis was performed for normally distributed data; spearman correlation was performed for abnormally distributed data. *P* value less than 0.05 was considered statistically significant.

## 3. Results

**Figure 1:**
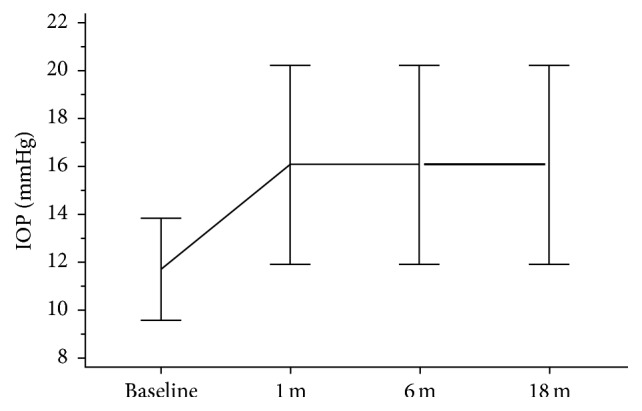
Mean IOP (in mmHg) at baseline, 1 month, 6 months, and 18 months postoperatively.

The study enrolled 54 eyes of 27 patients of mean age 29 ± 2.30 years. The mean baseline IOP of 11.69 ± 2.15 showed a statistically significant (*P* = 0.002) increase in IOP at 1 month postoperatively, which remained nearly unchanged at 6 and 18 months postoperatively, with mean value of 16.07 ± 4.12, 16.07 ± 4.10, and 16.07 ± 4.13, respectively, as shown in Figure 1.

One case presented with toxic anterior segment syndrome (TASS) 4 days postoperatively, with IOP of 47 mmHg, corneal edema from limbus to limbus, and UCVA of light perception. The condition was controlled with IV mannitol and topical steroids and antiglaucoma medications. IOP after 18 months was controlled to 23 mmHg on medications and reached 34 mmHg on no treatment.

After 1 month, the IOP was higher than 20 mmHg in 7 eyes (4 patients) during the study. At discharge, apart from the case with TASS syndrome, no eye had an IOP that was 10 mmHg or more above the preoperative IOP measurement 18 months postoperatively.

Of the 7 eyes with IOP higher than 20 mmHg, only 2 eyes with IOP of 22 and 23 mmHg required topical beta blockers, which controlled IOP over 1 month and stopped thereafter.

**Figure 2:**
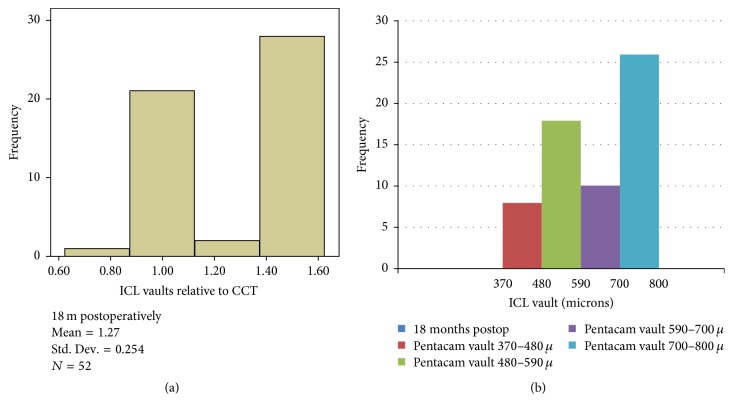
(a) The distribution of the pIOL ICL vaults (relative to CCT) of the 52 eyes over the crystalline lens, relative to the CCT, 18 months postoperatively. (b) The distribution of the Pentacam ICL vaults 18 months postoperatively.

The ICL vault was estimated clinically on slit lamp relative to the central corneal thickness and measured using Scheimpflug tomography in *μ*m. The proper amount of pIOL vault is considered to be identical to the thickness of the central cornea (approximately 500 *μ*m). The mean ICL vault measured at 1, 6, and 18 months postoperatively was 1.187 ± 0.279 (593 ± 135 *μ*m), 1.20 ± 0.275 (601 ± 133 *μ*m), and 1.274 ± 0.253 (637 ± 125 *μ*m), respectively. The variance was not statistically significant (*P* = 0.076) at 6 months and (*P* = 0.064) at 18 months, but there was a tendency for the pIOL vault to slightly increase over time. No eye had a postoperative pIOL vaulting grade of 0 (pIOL in contact with the lens) or 4 (excessive angular narrowing due to anterior displacement of the iris), both of which would have required pIOL explantation. The distribution of the pIOL vaults of the 52 eyes over the crystalline lens, relative to the CCT, 18 months postoperatively is shown in Figure 2(a), and Pentacam-measured vault (in *μ*) is shown in Figure 2(b).

Strong correlation was found between slit lamp (1x CCT) and Pentacam ICL vault in microns (*r* = 0.81).

**Figure 3:**
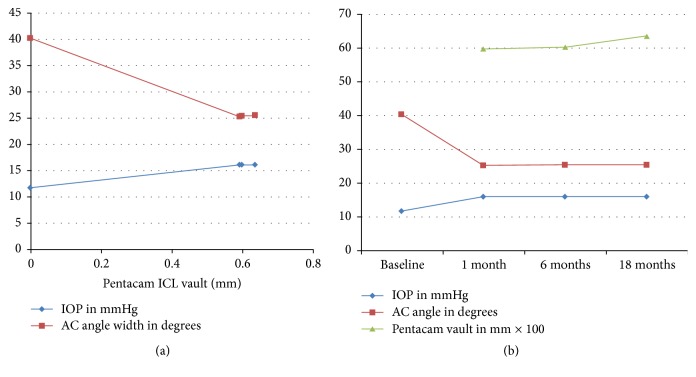
Changes in Pentacam AC angle width, Pentacam ICL vault, and IOP at different time points.

Increase in mean IOP, Pentacam ICL vault, and coincident decrease in AC angle width at the same time points is demonstrated in Figure 3.

**Figure 4:**
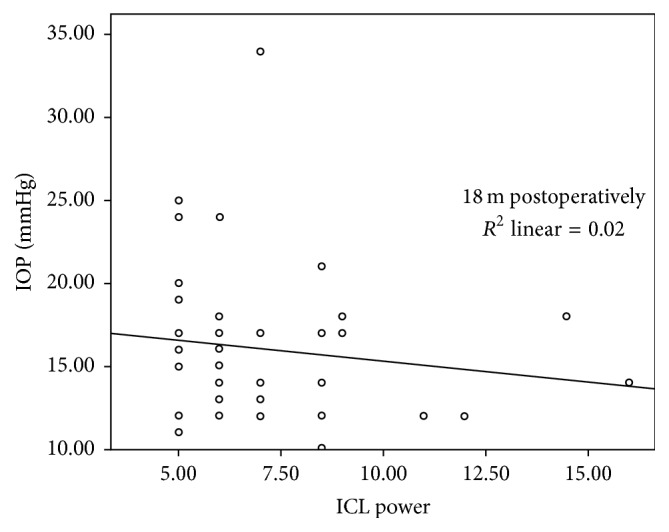
Correlation between mean ICL power and IOP 18 months postoperatively.

Mean ICL power (in diopters) of −6.85 ± 2.30 showed no correlation (*P* value of 0.131; *r* value −0.212) with mean IOP at 18 months, as shown in Figure 4.

**Figure 5:**
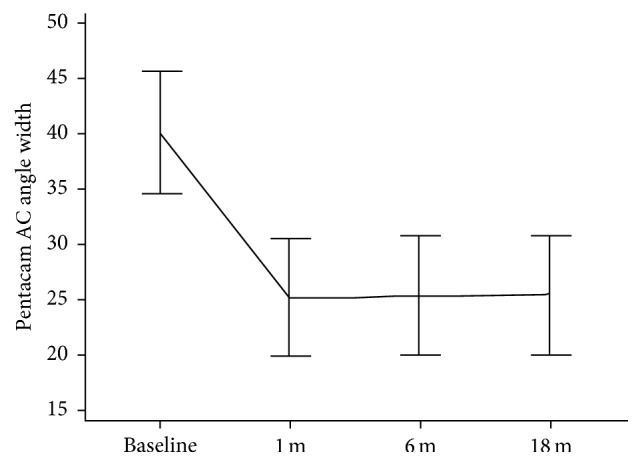
Mean Pentacam AC angle width at baseline and 1, 6, and 18 months postoperatively.

Pentacam AC angle width in degrees showed a statistically significant decrease at 1 (*P* = 0.025), 6 (*P* = 0.016), and 18 (*P* = 0.010) months postoperatively. Mean preoperative value of 40.14 ± 5.49 decreased to 25.280 ± 5.33, 25.469 ± 5.44, and 25.492 ± 5.38, at 1, 6, and 18 months, respectively, as shown in Figure 5.

Modified Van Herick limbal ACD grade at baseline (3.394 ± 0.069) decreased to 2.759 ± 0.065 (*P* = 0.741), 2.740 ± 0.066 (*P* = 0.689), and 2.692 ± 0.061 (*P* = 0.557) at 1, 6, and 18 months postoperatively, which is considered a nonstatistically significant decrease.

Pentacam-aided assessment of AC angle width showed no correlation with modified Van Herick grading system of limbal anterior chamber depth at all time points.

**Figure 6:**
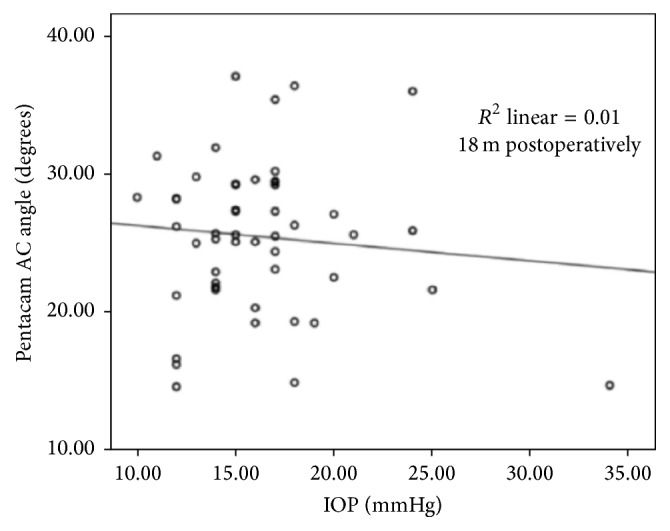
Correlation between Pentacam AC angle width (in degrees) and IOP (in mmHg) 18 months postoperatively.

Mean Pentacam AC angle width at 18 months showed no correlation with IOP, as shown in Figure 6.

Mean ICL vault showed moderate correlation with Pentacam AC angle width at 1 (*r* = −0.435) and 6 (*r* = −0.424) months and weak correlation (*r* = −0.271) at 18 months.

**Figure 7:**
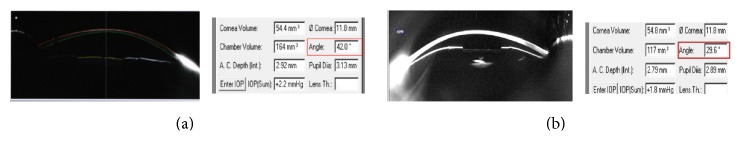
AC angle before (a) and after (b) ICL implantation in patient number 8.

Figure 7 demonstrates clearly AC angle width in patient number 8 of our case series, before and after ICL implantation.

## 4. Discussion

Intraocular pressure elevation following ICL implantation may be secondary to pupillary block, pigment dispersion, and steroid use [11]. Fujisawa et al. report that inserting an implantable Collamer pIOL alters the dynamics of the aqueous humor and results in IOP elevation [12].

Higueras-Esteban et al. in 2013 compared IOP following ICL implantation, in V4B and V4C groups. They found neither pupillary block nor significant IOP increase 3 months following V4C implantation in 18 eyes. They reported no intra- or intergroup significant difference in the mean IOP, between the conventional group and CentraFLOW group, with mean preoperative IOP values of 11.5 ± 2.8 mmHg in the V4B group and 11.9 ± 2.7 mmHg in the V4C group, compared to mean IOP after 3 months of 12.4 ± 1.8 mmHg in the V4B group and 13.8 ± 2.2 mmHg in the V4C group [13]. Despite lack of a peripheral iridectomy in the V4C group, physiologic aqueous flow was maintained with normal IOP levels. Spectral-domain OCT images showed ICL-iris contact in both V4B and V4C ICLs, without association with pigment dispersion [13].

Gonzalez-Lopez et al. monitored the mean IOP after V4C implantation. Baseline IOP was 14.6 ± 3.4 mmHg (range 8 to 26 mmHg) before surgery. Postoperatively, the mean IOP was 14.5 ± 4.6 mmHg (range 6 to 30 mmHg) at 1 day, 14.2 ± 4.2 mm Hg (range 6 to 29 mmHg) at 1 week, and 12.3 ± 3.4 mmHg (range 9 to 24 mmHg) at 1 month. No statistically significant alterations were detected over time after implantation (*P* > 0.2) [14].

In the current study, the mean baseline IOP of 11.69 ± 2.15 showed a statistically significant (*P* = 0.002) increase in IOP at 1 month postoperatively, which remained nearly unchanged at 6 and 18 months postoperatively, with mean value of 16.07 ± 4.12, 16.07 ± 4.10, and 16.07 ± 4.13, respectively. After 1 month, the IOP was higher than 20 mmHg in 7 eyes (4 patients) during the study. At discharge, apart from the case with TASS syndrome, no eye had an IOP that was 10 mmHg or more above the preoperative IOP measurement 18 months postoperatively.

Consistent with other authors [15], we attribute the early increase in IOP during the first month after surgery to the effect of postoperative inflammation, trabeculitis, and topical steroids, which may interpret the statistically significant increase in IOP 1 month postoperatively in the current study. Furthermore, highly myopic patients are more prone to steroid-related increases in IOP, especially with high intraocular penetration like topical prednisolone acetate.

Despite the lack of a strong correlation between increased postoperative IOP with decreased AC angle width or increased ICL vault throughout the 18 months of follow-up, Figure 3 interpretation may attribute the elevated IOP following ICL implantation to the coincident decreased AC angle width at the same time points.

In the current study, the presence of the 0.36 mm AquaPORT with its known fountain effect on back of Collamer lens may explain the mild increase in ICL vault throughout the 18 months of follow-up. However, the presence of a central hole did not preclude the reported IOP elevation and reduced AC angle width at 1, 6, and 18 months postoperatively.

In our study, the ICL vault variance was not statistically significant (*P* = 0.076) at 6 months and (*P* = 0.064) at 18 months, but there was a tendency for the pIOL vault to slightly increase over time. Mean ICL vault showed moderate correlation with Pentacam AC angle width at 1 (*r* = −0.435) and 6 (*r* = −0.424) months and weak correlation (*r* = −0.271) at 18 months.

ICL vaulting measurements in our study disagreed with Kamiya et al. who reported nonsignificant decrease over months of follow-up. Increase in follow-up vault in our study may be described as dynamic vaulting, possibly explained by the fountain effect, secondary to a constant aqueous-pushing force to overlying Collamer lens. However, Kamiya et al. assumed that the presence of an artificial hole does not significantly affect the amount of the pIOL vault because of the continuous pressure exerted by the back surface of the iris on the pIOL [16].

Despite the statistically significant decrease in AC angle width following V4C ICL implantation, mean Pentacam AC angle width 18 months following surgery showed no correlation with increase in IOP. However, long-term follow-up of AC angle width should be performed to detect potential angle closure. Besides, we recommend the addition of AC angle width (in degrees) to the preoperative data required by the STAAR Company during online lens calculation and ordering.

In a study by Chung et al., one-month postoperative angle opening distance values were significantly smaller than preoperative values by 31.8% (*P* < 0.001), but no significant progressive changes were observed thereafter [17].

In our study, late postoperative increase in ICL vault and decrease in AC angle width are not necessarily the result of overestimated preoperative W-W and subsequent large lens diameter calculation.

Instead of resting on ciliary sulcus, ICL footplate haptics might be supported by the ciliary body that could lead to narrowing of the AC angle [17]. Besides, age-related increase in ciliary muscle thickness might alter the late postoperative ICL lens position by a forward shift of the ICL, with subsequent vault increase in the later periods after ICL implantation [18], which may add a possible explanation to the vault increase in the current study.

No cases of pupillary block due to obstruction of the central port were reported in the current study or in any of the few previously published studies (theoretical and in vivo) [14].

The Collamer lens power is a reflection of its thickness that—theoretically—may result in AC shallowing and IOP elevation. However, in our study, mean ICL power (in diopters) of 6.85 ± 2.30 showed no correlation (*P* value of 0.131; *r* value −0.212) with mean IOP at 18 months.

One of the advantages in this study was the dynamic noncycloplegic vault measurements, which did not interfere with the accommodation-induced changes. Since the pIOL optic remains in contact with the back surface of the iris, the latter pushes the pIOL toward the crystalline lens before mydriasis, and the anterior surface of the crystalline lens shifts posteriorly after mydriasis. Accordingly, the amount of the pIOL vault may be affected by pupil dilatation.

The limitations of this study include the lack of a comparative study group with conventional posterior chamber phakic lens and the lack of correlation of ICL vault with pupil diameter before and after ICL insertion. Besides, vaulting and AC angle measurements would have been better triple-checked with an additional imaging modality like anterior segment OCT that permits high-resolution cross-sectional anterior segment imaging with excellent reproducibility of measurements by using the interference profile of the reflections from the cornea, iris, and crystalline lens. In addition, Pentacam-aided assessment of AC angle width in our study showed no correlation with Van Herick grading system of limbal anterior chamber depth.

To our knowledge, this is the first study to assess the correlation between ICL vault, AC angle width, and intraocular pressure following V4C lens implantation, with follow-up up to 18 months postoperatively.

A long-term careful observation is required to compare the pIOL vault with and without cycloplegia in both V4B and V4C Collamer lenses, with correlation to AC angle width.

In summary, our noncomparative study demonstrated that implantation of Visian ICL with a central hole resulted in a decrease in AC angle width and increase in IOP, within acceptable physiological values at all time points, with zero incidence of pupillary block-angle closure glaucoma.

## Figures and Tables

**Table 1:** Limbal anterior chamber depth grading system.

Van Herick grade	Limbal anterior chamber depth: corneal section thickness (fraction)	Limbal anterior chamber depth (percentage of corneal section thickness)
0	0 (closed)	0%
1	<1/4	5% 15%
2	1/4	25%
3	1/4 to 1/2	40% 75%
4	1 or >1	≥100%
